# Primary ovarian endometrioid stromal sarcoma presenting with reno-ureteral colic

**DOI:** 10.1186/s12905-022-02046-9

**Published:** 2022-12-03

**Authors:** Ana Isabel Bueno Moral, José Carlos Vilches Jiménez, Carmen Martínez Bañón, Manuel Jesús Díaz Huesca, Miriam Valenzuela González, Jesús S. Jiménez López

**Affiliations:** 1 Department of Obstetrics and Gynaecology, Regional University Hospital, 29011 Málaga, Spain; 2 Departament of Gynaecology and Gynaecological Oncology, Hospital Quiron Salud, Málaga, Spain; 3 Departament of Pathological Anatomy, Regional University Hospital, 29011 Málaga, Spain

**Keywords:** Ovarian sarcoma, Reno-ureteral colic, Ovarian cancer, Endometrial stromal sarcomas

## Abstract

**Background:**

Endometrioid Stromal Sarcomas are an infrequent group of mesenchymal tumors that we must take into account in the differential diagnosis despite representing only 0.2% of tumors of the female genital tract, as they can go unnoticed until advanced stages.

**Case presentation:**

Fifty-fourth year-old woman referred from the Urology department due to incidental finding of adnexal mass in MRI during examination after renoureteral colic, in the case of a 50 mm solid cystic mass in LE. MT were within the normal range, and the CT scan observed this mass in contact with the left ureter. The surgery was completed with hysterectomy and contralateral adnexectomy without incident and chemotherapy treatment was not added. The pathological result was ovarian tissue with low-grade endometrial sarcoma. Currently, after two years of follow-up, the patient remains stable without any recurrence of disease.

**Conclusions:**

Endometrioid stromal sarcomas are rare tumors that originate in the endometrial stroma, the ovarian location being rare. Management lies in surgical treatment, and adjuvant therapy is sometimes necessary in advanced stages.

## Background

Endometrioid Stromal Sarcomas (ESS) are rare mesenchymal tumours that can occur in women across a wide range of age, from puberty to menopause, taking place around a median age of 50 years, and that represents only 0.2% of all female genital tract tumors and 15–26% of primary uterine sarcomas [1–8].

An extrauterine location is extremely rare with ovaries being the most common location, although another type of locations has been reported involving the intestinal wall, peritoneum, pelvis and vagina [2, 5–7]. The clinical presentation is sometimes non-specific and heterogeneous, with patients having symptoms related to the location and size of the tumor or being asymptomatic, which results in a late diagnosis in advanced stages.

The main treatment for this type of tumour is surgery, although adjuvant treatment, chemotherapy and radiotherapy, may be of benefit in advanced stages [2, 4]. There is also a role for hormonal therapy in tumours expressing high levels of progesterone receptors [3, 7].

## Case presentation

A 54-year-old woman with a personal medical history of hypertension only who is receiving hydrochlorothiazide therapy. No other pathologies, no drug allergies and no previous surgeries. Regarding her obstetric and gynaecological history, she has had two deliveries and one spontaneous miscarriage, as well as established menopause one year and half ago. The last gynaecological examination with transvaginal ultrasound was performed 3 months earlier and was reported to be normal.

The patient was referred to the gynecological outpatients department from the urology unit due to an incidental finding of adnexal mass on MRI during a medical examination performed after a renal-ureteral colic.

**Fig. 1:**
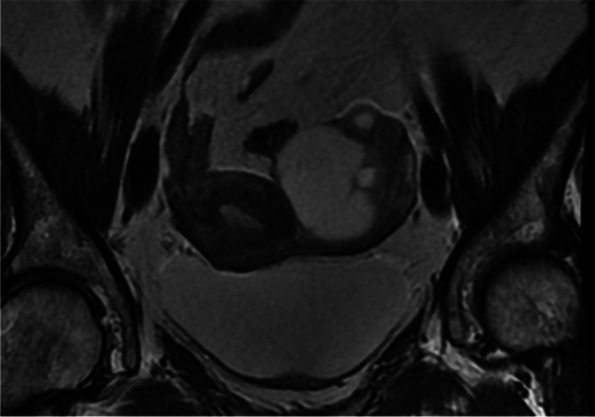
MRI T2-weighted image of solid-cystic lesion

The MRI report included the following: solid-cystic mass in the left ovary with a 50 mm size, anterior cystic component and a heterogeneous solid pole on the posterior wall visualised on the contrast-enhanced ultrasound, having irregular contours and diffusion restriction (Fig. 1).

The abdomen was soft, depressible and painless to palpation with no palpable masses present. The vagina and external genitalia were normal in appearance, and the cervix was macroscopically normal. Bimanual examination revealed a mobile cyst formation involving the left adnexa, but it was hard and tender palpation, and with regular contours.

**Fig. 2:**
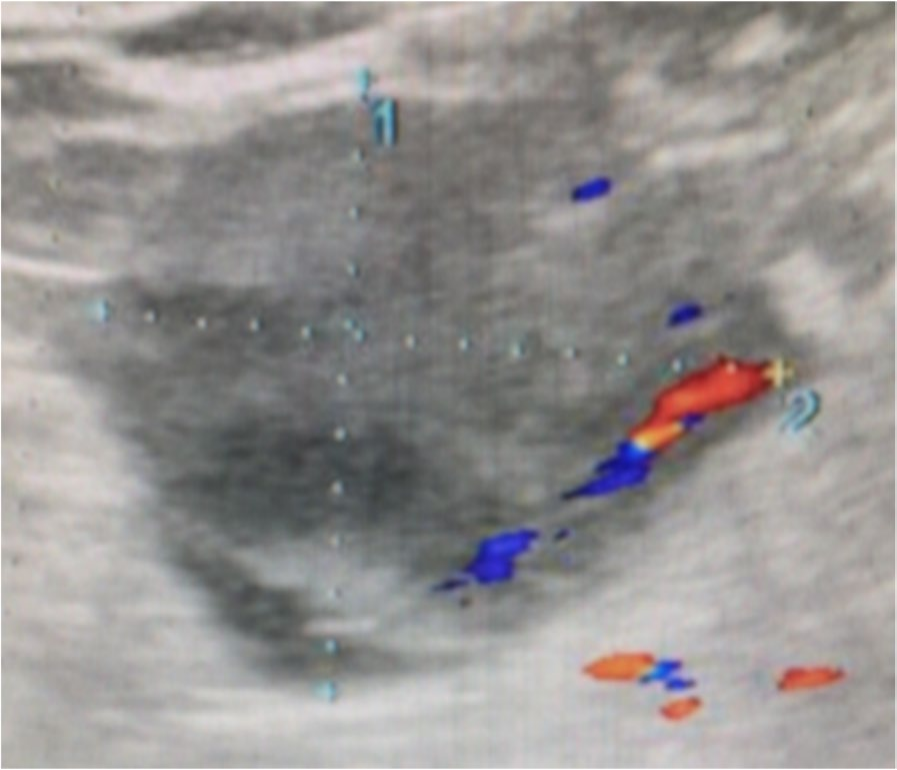
Ultrasound image of solid-cystic lesion

An ultrasound scan showed an anteverted uterus measuring 59 × 33 mm with a 5-mm-thickened endometrium at the expense of a formation with hyper reflectivity compatible with an endometrial polyp. The right adnex was normal in appearance. In the left ovary, it was observed a multiloculated (5 loculi) solid-cystic mass measuring 50 × 46 mm, with anechoic contents and regular contours, thin septa, no papillae, with crescent sign and a vascular score of 1 compatible with a borderline lesion (Fig. 2). No free fluid in the pouch of Douglas.

**Fig. 3:**
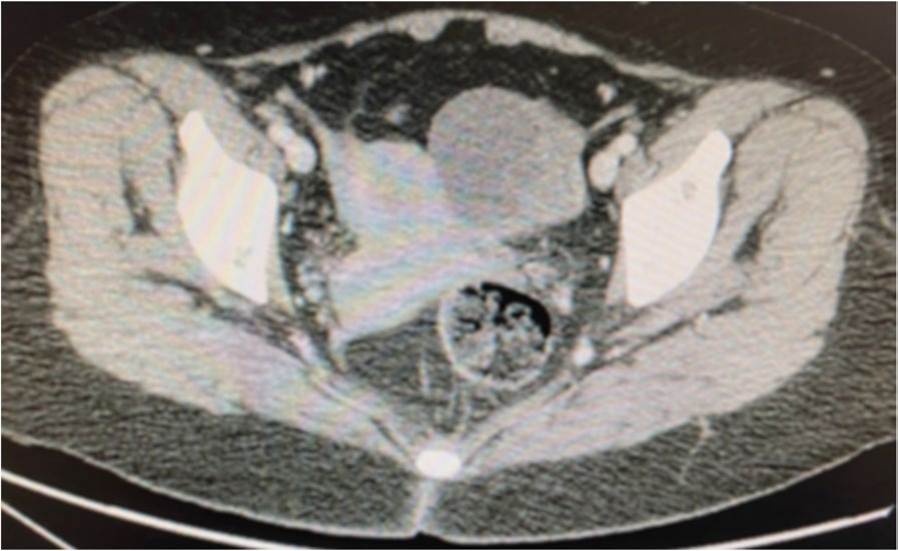
Abdominopelvic CT scan showing the above-described solid-cystic formation measuring 50–60 mm

After diagnosis, tumor markers and an abdominopelvic CT scan were requested. Tumor markers (TM) were in the normal range (CA 125 10, CA 19.9 6, CEA 2, HE4 45 and ROMA index 8) and the CT scan was reported as showing a heterogeneous solid-cystic image measuring 55 × 59x64 mm located in the left parauterine region of potential left adnexal origin that seems to be in contact with the left ureter (Fig. 3).

The case was discussed in a committee comprising specialists in gynecology and gynecology oncology, and it was decided along with the patient to perform an exploratory laparotomy.

A midline infra-umbilical laparotomy was performed. During surgery it was observed the above-mentioned solid-cystic mass measuring 60 mm in the left ovary, with smooth surface, firmly attached to the peritoneum of the ovarian fossa and loosely to the colon. No infiltration or compromise of the ureter or vascular structures was observed. The rest of the examination of the abdominopelvic cavity was normal. An intraoperative biopsy was performed and no malignant features were found, so the surgery was completed with total hysterectomy and contralateral adnexectomy with no incidents.

The postoperative course was favourable and the patient was discharged after four days without complications.

Peritoneal lavage cytology was examined in the pathological report and results were negative. There was present a moderate chronic cervicitis, an inactive endometrium and an endometrial polyp inside the uterus. The right adnex was normal and there was a fragment of ovarian tissue with low-grade endometrial sarcoma in the left ovary.

**Fig. 4:**
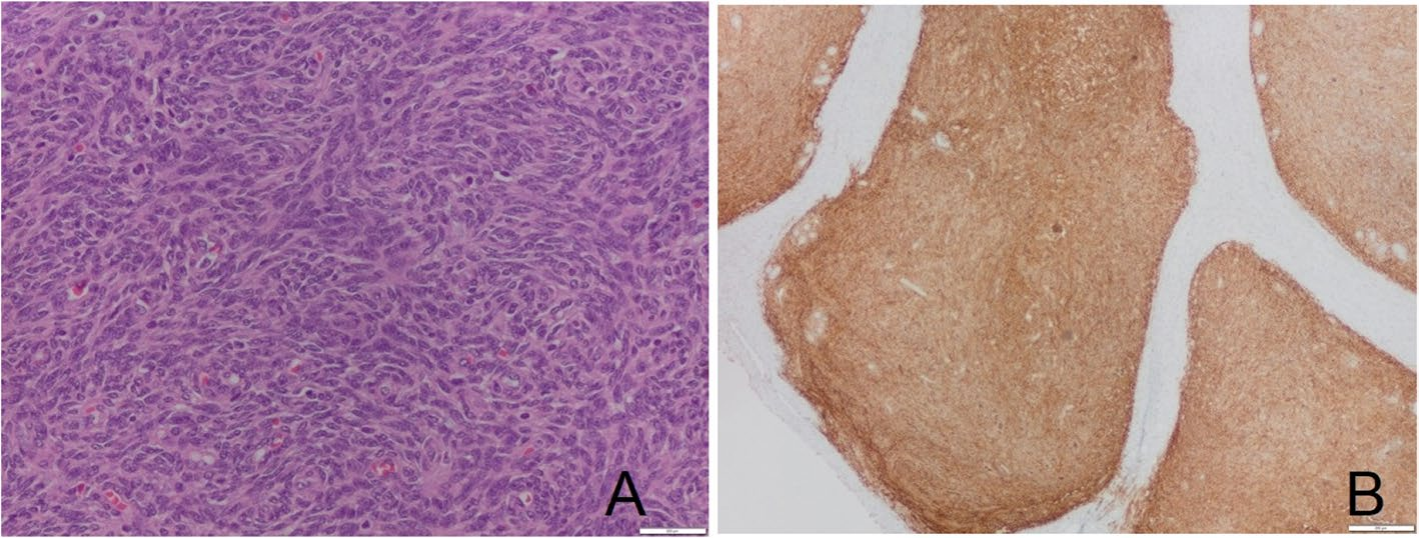
Histologic aspects of the ovarian tumor. Hematoxylin and eosin stain × 20 (**A**) and inmmunohistochemical features of the tumor with tumor cells positive for CD 10 (**B**)

The pathological report revealed nests of cells with uniform oval and round shape in fragments of the left ovary, with very scant cytoplasm, no cytologic atypia and a low mitotic index accompanied by abundant small vessels (Fig. 4a).

At immunohistochemistry, tumor cells were positive for CD 10, desmin and for estrogen and progesterone receptors (ER, PR). They were negative for smooth muscle actin (SMA) and alfa-inhibin. Proliferative index < 5% (Fig. 4b).

The case was presented to the tumor committee of our hospital ( composed by pathologists, medical and radiation oncologists, radiologists and gynecologists) and it was agreed to follow up by gynaecology without the need for adjuvant treatments. After two years of follow-up care in the oncology outpatients department, the patient is asymptomatic and is having routine checkups with normal TM and six-monthly CT scans that show no findings.

## Discussion and conclusion

Endometrioid stromal sarcomas are an infrequent group of tumors that arise in the endometrial stroma and primarily affect the uterus. These show differentiation in the proliferative stage, which is similar to that of the endometrial stroma but without evidence of a uterine origin, being the ovary the most frequent location [2]. In our case, the patient was 54 years old and had no gynecologic history of interest.

The latest WHO classification from 2014 divided ESS into 4 subgroups based on clinical and pathological characteristics: endometrial stromal nodule (ESN), low-grade endometrial stromal sarcoma (LG-ESS), high-grade endometrial stromal sarcoma (HG-ESS) and undifferentiated uterine sarcoma. The most frequent group is the low-grade ESS with a 5-year survival rate of 90% in the early stages, although it presents a high recurrence rate [2]. The other groups have lower survival rates being around 30% for high-grade endometrial stromal sarcoma and undifferentiated uterine sarcoma [9].

Therefore, its diagnosis includes different techniques such as the use of imaging tests (contrast-enhanced T2-weighted MRI) or the positive expression of tumor markers, such as CD-10, vimentin or estrogen/progesterone receptors by immunohistochemistry [2, 5, 7]. However, the lack of specificity of these tests as well as the potential fluctuation of CA-125 levels within normal range suggests the surgical approach to posterior histological analysis to be the best diagnostic and treatment option. In this case, hysterectomy and bilateral salpingo-oophorectomy procedures are the standard of care [4].

The basic treatment for the initial disease includes hysterectomy and bilateral salpingo-oophorectomy [2, 4, 7]. Regarding the adjuvant therapy (hormonal therapy, chemotherapy and radiotherapy), despite the fact that there are no standardized protocols and no clinical trials that have been able to confirm if there are any survival benefits due to the tumor rarity and the disease heterogeneity, it should be considered for patients with advanced-stage cancer (chemotherapy and radiotherapy combination) and expressing high levels of progesterone receptors (hormonal therapy) [2–4, 7]. The use of additional therapies depends on whether the excised tumour is low or high grade and whether sarcoma components may remind of a fibrosarcoma or a endometrioid stromal sarcoma. The latter could be potentially treated with progesterone, radiotherapy or both, while chemotherapy may be indicated for high-grade fibrosarcomas.

This tumor can lead to local recurrence or even distant metastasis a few years later after the initial diagnosis. It has been estimated that they usually recur in about 50% of cases [2, 10]. The extra-ovarian dissemination and a high tumor grade are associated to a high recurrence rate, even at stage I. It suggests that an age below 53 years, a tumor rupture and a high tumour grade are key predictors of disease recurrence, particularly at stage I. It seems that the premenopausal status of the patient, and therefore the hormonal status, might play a role in these tumors progression [10].

After reviewing the literature, we have found a total of 91 cases, of which 41 have been treated with surgery only and 41 with a treatment-associated surgery (chemotherapy or hormonal therapy), whereas only 9 cases were treated with radiotherapy.

Ovarian adenosarcomas have a similar prognosis to the homonymous uterine sarcoma and have a tumour recurrence in 50% of patients, which occurs almost always within the first 5 years after surgery [1, 2, 10]. The 5-year overall survival rates of patients with FIGO stage I have been higher than 90% and 30% in patients with FIGO stage II, whereas patients with FIGO stage III and stage IV have worse survival rates, that is, 50% [4, 11].

The appearance of this type of ovarian tumor may be related to the previous presence of endometriosis. Although the pathogenesis is not clear, there seems to be a risk of malignization of these endometriotic foci. Nevertheless, the presence of endometriosis is considered a favorable factor since these patients have a higher DFS than patients without endometriosis.

In cases of extrauterine tumor without endometriosis (as in the case of our patient) the origin may arise from pluripotent mesothelial and mesenchymal mesenchymal cells of the pelvic cavity.

Most patients at stage I have at least one relapse. High recurrence rates are associated with patients aged under 53 years with stage I disease, a high-grade tumour rupture and sarcomatous overgrowth. The time from diagnosis to the first recurrence has been estimated to be 0.4 to 6.6 years (mean: 2.6 years; median: 2.2 years). Distant metastatic lesions, if any, are usually preceded by intra-abdominal recurrences. A recurrence can occur as a low-grade or high-grade adenosarcoma or as a sarcoma of variable grade [12, 13].

In our case, the patient was presented to an Oncology Committee and, given that it was a low-grade tumor confined to the ovary, it was decided not to prescribe adjuvant therapy but to follow her closely. Currently, after two years of follow-up, the patient remains asymptomatic and stable with no evidence of recurrence.

As shown above, there are several strategies used in the management of this disease entity, partly justified by its rarity and the absence of specific protocols laying down solid standards for the disease management. Although no significant differences were found in terms of results, this challenge is one of the main objectives of future research at ESS.

## Data Availability

All data generated or analysed during this study are included in this published article.

## Abbreviations

MRI: Magnetic Resonance Imaging
TM: Tumour markers
CA 125: Cancer antigen 125
CA 19.9: Cancer antigen 19.9
HE-4: Human Epididymal Protein
TC: Computed tomography
DFS: Disease free survival
